# Editorial: Cerebrovascular and Neurodegenerative Diseases - New Insights Into Molecular Cell Biology and Therapeutic Targets

**DOI:** 10.3389/fneur.2019.01322

**Published:** 2019-12-13

**Authors:** Sikha Saha, Sriharsha Kantamneni

**Affiliations:** ^1^Leeds Institute of Cardiovascular and Metabolic Medicine, University of Leeds, Leeds, United Kingdom; ^2^School of Pharmacy and Medical Sciences, University of Bradford, Bradford, United Kingdom

**Keywords:** neurodegenerative disease, molecular biology, stroke, middle cerebral artery and common carotid artery occlusion, cerebrovascular disease, therapeutics

There is a significant gap in our understanding of the molecular and cellular biology of cerebrovascular and neurodegenerative diseases to identify new therapeutic targets and develop diagnostic tools to better understand the disease progression and treatment. The cellular and molecular events linking cerebrovascular pathology and neurodegeneration are also not fully understood. The research articles and review papers published in this topic aim at a multifaceted approach to evaluating recent progress in our understanding some of the underlying molecular mechanisms of disease process and potential therapeutics targeting these diseases. Here we summarize the contributing articles to our topic conveying the aim of the pertaining research. The articles in this Research Topic highlight the challenges inspiring future research to address some of the questions and to exploit new opportunities for development of novel therapeutics for cerebrovascular and neurodegenerative diseases.

Smith-Dijak et al. studied the effects of pridopidine, a drug that enhances brain derived neurotrophic factor (BDNF) signaling through stimulation of the sigma-1 receptor (S1R) and S1R agonist, in cortical neurons obtained from a mouse model of Huntington disease (HD). Several pathways implicated in synaptic functions are dysregulated in HD, including BDNF and calcium signaling. The data provide evidence for restoration of synaptic plasticity that maintain the stability of neuronal and synaptic function required for new learning and cognitive function. The results suggest a potential new direction for developing therapy to mitigate cognitive deficits in HD and may provide new avenues for neuroinflammation-related disorders treatment.

Agouni et al. provided a comprehensive analysis of circulating extracellular vesicles (EVs) from vascular wall, blood, and immune cells in transient ischemic attacks (TIA) and acute ischemic stroke (AIS) patients from Southeast Asia and the Middle East. This study showed that EVs of various origins, especially those associated with endothelial cell injury and platelet activation, are increased in TIA and AIS patients. The levels of EV continue to be high for up to 30-days post-attacks indicating a sustained cellular activation, which may be associated with an increased risk of recurrence of acute events in this population.

D'Angelo et al. carried out a review on antiphospholipid syndrome (APS) and multiple sclerosis (MS). APS and MS are both considered as anti-lipid autoimmune diseases with specific pathophysiological mechanisms and events. Isolated neurological APS represents a significant diagnostic challenge, as epidemiological, clinical, and neuroimaging features may overlap with those of MS. The review draws attention to the clinical relevance of diagnosing isolated neurological APS and suggests that prompt and accurate diagnosis and treatment of APS with anti-aggregant and anticoagulant could be vital to prevent or reduce APS-related morbidity and mortality.

Wei et al. examined the cellular mechanisms mediating the neuroprotective effects of Homer1a, a short form of a scaffold protein, which is upregulated in rat cortical neurones following oxygen and glucose deprivation (OGD) mimicking ischemia-reperfusion (I/R) injury. The results showed that overexpression of Homer1a reduced OGD-induced lactate dehydrogenase (LDH) release, cell death, and mitochondrial dysfunctions in cultured cortical neurons. Homer1a also protects against OGD-induced injury by preserving mitochondrial function through inhibiting the protein kinase R-like endoplasmic reticulum kinase (PERK) pathway. In addition, mitochondrial protection of Homer1a was blocked by the ER stress activator tunicamycin (TM) suggesting that Homer1a may be a promising target of protecting neurons from cerebral injury.

Yang et al. examined the effect of cyclooxygenase (COX2)/prostaglandin D2 (PGD2)-related autophagy on brain injury in diabetic rats suggesting that the COX2-PGD2 pathway is a potential therapeutic target for diabetic brain injury.

Xu et al., reported that low-moderate ethanol consumption may prevent ischemic stroke and reduce brain cerebral ischemia/reperfusion injury (I/R) by suppressing inflammation, whereas heavy alcohol consumption may induce ischemic stroke and worsen brain I/R injury by aggravating inflammation.

He et al. found that smilagenin, a steroidal sapogenin from traditional Chinese medicinal herbs, can have neuroprotective effect on dopaminergic neurons in a chronic mouse model of Parkinson's disease (PD) suggesting that this drug could prevent the impairment of dopaminergic neurons in PD.

Zhang et al. demonstrated that Naringenin (NAR), a grapefruit flavonoid promoted microglia M1/M2 polarization, thus conferring anti-neuroinflammatory effects via the inhibition of mitogen-activated protein kinase (MAPK) signaling activation. These findings provide new alternative avenues for neuroinflammation-related disorders treatment.

Hao et al. showed that heterozygous loss of activin receptor-like kinase 1 (Alk1) can lead to hereditary hemorrhagic telangiectasia, which is a vascular disease characterized by direct connections between arteries and veins leading to arteriovenous malformations (AVMs). The results of the study suggest that Alk1 induces the formation of sporadic human cerebral AVMs through affecting migration and proliferation of endothelial cells combined with vascular endothelial growth factor A.

Li et al. developed a flow cytometry protocol to identify microglia and monocyte-derived macrophages from mouse intracerebral hemorrhagic (ICH) stroke model induced by collagenase or blood injection. The authors also combined magnetic-activated cell separation system that allows eight tissue samples to be assessed together. This protocol represents a very important tool for biological functions of microglial and monocyte-derived macrophage in ICH stroke and related brain diseases.

Jin et al., showed that glucose-regulated protein (GRP78) a chaperone protein located in the endoplasmic reticulum (ER) is involved in the neuroglial response to neurotoxic insult in rats induced by mitochondrial toxin 3-nitropropionic acid (3-NP), which selectively damages striatal neurons. These data provide novel insights into the phenotypic and functional heterogeneity of GRP78-positive cells within the lesion core and the involvement of GRP78 in the activation/recruitment of activated microglia/macrophages and blood-brain-barrier impairment in response neurotoxic insult.

Song et al. reported that the knock down of myosin light chain kinase, a key enzyme in smooth muscle cell contraction, in human brain smooth muscle cells (SMCs) caused effects similar to those observed in cultured SMCs from intracranial aneurysm patients. These results indicate that myosin light chain kinase plays an important role in maintaining smooth muscle contractility, cell survival and inflammation tolerance and is crucial to the normal function of intracranial arteries.

Hoyk et al. showed elevated serum triglyceride levels, changes in functional and morphological gene expressions and blood brain barrier dysfunction in transgenic mice overexpressing the human APOB-100 protein, a mouse model of human atherosclerosis suggesting that these transgenic mice could be a useful model to study the link between cerebrovascular pathology and neurodegeneration.

Yu et al. reported that injection of hydroxysafflor yellow A (HSYA), a major active chemical component of the safflower via carotid artery improves cognitive impairment and synaptic plasticity in a rat model of stroke induced by middle cerebral artery occlusion.

Chen et al. showed that Gap19, a selective Connexin 43 (Cx43) -hemi -channel inhibitor, produces neuroprotective effects in cerebral ischemia/reperfusion injury induced by middle cerebral artery occlusion in mice via suppression of Cx43 and Toll-like receptor 4 (TLR4) mediated signaling pathways.

Chang et al. developed a new *in vitro* cerebral micro-bleed model to study the interactions between brain endothelial cells and red blood cells exposed to oxidative stress. Their findings demonstrate that erythrophagocytosis mediated by the brain endothelial monolayer and the passage of iron-rich hemoglobin and RBC, may be involved in the development of cerebral microbleeds that are not dependent on disruption of the microvasculature.

Faustino-Mendes et al. reviewed the current experimental models of immature ischemic brain and highlighted the need for new multifactorial experimental models to attain more efficient therapies to treat this complex vascular condition and related long-term conditions.

Chen et al. showed that the compound 22a, a promising neuroprotective compound derived from tetramethylpyrazine and widely used as active ingredient of traditional Chinese medicine, effectively prevented glutamate-induced excitotoxicity in cerebellar granule cells (CGNs) via involvement of the PI3K/Akt and PGC1α/Nrf2 pathways suggesting that this compound might be useful in preventing neuronal death from ischemic stroke.

Du et al. tested a hypothesis that chemokine interleukin (IL) eight released by astrocytes and C-X-C motif chemokine receptor 1 (CXCR1) in neurons are involved in neuronal apoptosis induced by methamphetamine (METH), a widely abused illicit drug, which can cause dopaminergic neuron apoptosis and astrocyte-related neuroinflammation. The results suggest that CXCR1 may be a potential target for METH-induced neurotoxicity therapy.

Lu et al., aimed to explore the protective effects of rosuvastatin, a 3-hydroxymethyl-3-methylglutaryl coenzyme A (HMG-CoA) reductase inhibitor, against haemorrhagic transformation (HT) after recombinant tissue plasminogen activator (rt-PA) treatment in a mouse model of experimental stroke. The beneficial effects are related to inhibition of the inflammation-related nuclear factor kappa B (NF-κB) and mitogen-activated protein kinase (MAPK) pathways.

Wang et al. showed that mild ER stress (“preconditioning”) induced by tunicamycin (TM), can alleviate LPS-induced astrocytic activation and BBB disruption. Their findings provide a better understanding for the regulatory role of ER stress in neuroinflammation and indicate that mild ER stress might have therapeutic value for the treatment of neurodegenerative diseases.

Yang et al., found that adapentpronitrile, a new adamantane-based dipeptidyl peptidase-IV (DPP-IV) inhibitor significantly ameliorated neuronal injury and decreased amyloid precursor protein (APP) and amyloid beta (Aβ) expression in the hippocampus and cortex of rat model of diabetes fed with high fat diet. These authors showed that adapentpronitrile protected against diabetic neuronal injury by inhibiting mitochondrial oxidative stress and the apoptotic pathway.

Jang et al. reported intrastriatal injection of adeno-associated viral vector serotype DJ containing N171-82Q mutant huntingtin *(HTT) gene* to juvenile mice produced Huntington's disease (HD)-like symptoms including mutant *HTT* aggregation, neurodegeneration, and Neuroinflammation. The authors suggested that this model will a useful tool to better understand neuropathological mechanisms of HD and develop new therapeutics for this disease.

Hao et al. established a stable method for the isolation of endothelial cells (ECs) from human cerebral arteriovenous malformation tissues, which play an important role in the manifestation and development of cerebral vascular malformation as well as haemorrhagic stroke and thrombogenesis. The protocol can also be adapted for other vascular diseases.

He et al. demonstrated that rosuvastatin treatment significantly increased neurite outgrowth in cortical neurons after oxygen-glucose deprivation (OGD)-induced damage, reduced the generation of reactive oxygen species, protected mitochondrial function and elevated the ATP levels via Notch1 pathway. These findings highlight Notch1 signaling as important player and novel therapeutic target in promoting brain plasticity.

In summary, the present Research Topic encompassed several cutting-edge techniques and models for investigating the cellular and molecular mechanisms of cerebrovascular dysfunction and neurodegeneration. Together, they provide a framework for understanding the way some of the diseases progress and potential pathways for therapeutic interventions. We acknowledged that collection of articles in a single topic cannot deal with this extremely vast subject characterized by complex conditions such as cerebrovascular dysfunction and neurodegeneration. The topics addressed, however, help developing clear ideas, not only in terms of recent studies but also the unmet needs for future research in these areas. We trust that the papers assembled in this Research Topic will prove useful in encouraging and stimulating future progress in research related to cerebrovascular and neurodegenerative diseases.

## Author Contributions

SS prepared the editorial and both SK and SS edited the final version. SS and SK made substantial contributions to the review and approved the manuscripts accepted on this topic.

### Conflict of Interest

The authors declare that the research was conducted in the absence of any commercial or financial relationships that could be construed as a potential conflict of interest.

